# Cancer biology deciphered by single-cell transcriptomic sequencing

**DOI:** 10.1007/s13238-021-00868-1

**Published:** 2021-08-17

**Authors:** Yanmeng Li, Jianshi Jin, Fan Bai

**Affiliations:** 1 Biomedical Pioneering Innovation Center (BIOPIC), School of Life Sciences, Peking University, Beijing, 100871 China; 2 RIKEN Center for Biosystems Dynamics Research (BDR), 6-2-3, Furuedai, Suita, Osaka Japan; 3 Beijing Advanced Innovation Center for Genomics (ICG), Peking University, Beijing, 100871 China

**Keywords:** single-cell transcriptomic sequencing, tumor microenvironment, cancer

## Abstract

Tumors are complex ecosystems in which heterogeneous cancer cells interact with their microenvironment composed of diverse immune, endothelial, and stromal cells. Cancer biology had been studied using bulk genomic and gene expression profiling, which however mask the cellular diversity and average the variability among individual molecular programs. Recent advances in single-cell transcriptomic sequencing have enabled a detailed dissection of tumor ecosystems and promoted our understanding of tumorigenesis at single-cell resolution. In the present review, we discuss the main topics of recent cancer studies that have implemented single-cell RNA sequencing (scRNA-seq). To study cancer cells, scRNA-seq has provided novel insights into the cancer stem-cell model, treatment resistance, and cancer metastasis. To study the tumor microenvironment, scRNA-seq has portrayed the diverse cell types and complex cellular states of both immune and non-immune cells interacting with cancer cells, with the promise to discover novel targets for future immunotherapy.

## INTRODUCTION

Cancer is a genetic disease that continues to seriously threaten human health. Its evolutionary progression comprises the acquisition and accumulation of oncogenic somatic mutations and non-genetic alterations, which may lead to unrestrained proliferation, invasiveness, and treatment resistance of tumor cells (Weinberg, 2002; Pantel and Brakenhoff, 2004; Merlo et al., 2006; Weis and Cheresh, 2011; Wan et al., 2013; Lambert et al., 2017; Maley et al., 2017). A comprehensive understanding of the molecular mechanisms underlying carcinogenesis is critical for diagnosis, drug development, clinical trial design and therapeutic strategy selection. In the past decade, next-generation sequencing (NGS) has advanced our understanding of cancer biology in many fields such as cancer genomics and transcriptomics (Cancer Genome Atlas Research, 2012a, b, 2014, 2016; Network, 2012a, b; Garraway and Lander, 2013; George et al., 2015; Wang et al., 2016; Nakagawa and Fujita, 2018), tumor evolution (Fearon and Vogelstein, 1990; Gerlinger et al., 2012; Sottoriva et al., 2015; Williams et al., 2016), intra-tumor heterogeneity (Gerlinger et al., 2012; Burrell et al., 2013; McGranahan and Swanton, 2017; Turajlic et al., 2019), and treatment resistance (Murugaesu et al., 2015; Swanton and Govindan, 2016; Chen and Mellman, 2017; Turajlic and Swanton, 2017; Berger and Mardis, 2018; Binnewies et al., 2018). Although great achievements have been made, most conventional analyses and datasets are from bulk samples, which average the molecular features of cells in a tumor sample.

Tumors are increasingly viewed as complex ecosystems, in which heterogeneous cancer cells interact with their microenvironment composed of, but not limited to, diverse immune, endothelial, and stromal cells (Chen and Mellman, 2017; Binnewies et al., 2018; Valkenburg et al., 2018). Emerging evidence show that not only the cancer cells themselves but also the cells in the microenvironment shape the biological and clinical behavior of a tumor. This insight suggests that bulk profiling of tumors may mask the cellular diversity and is hard to study the molecular mechanisms of different cell types in a tumor which may contribute to carcinogenesis in distinct ways. Therefore, to handle the cell heterogeneity in tumors, the recently developed single-cell sequencing technologies (Tang et al., 2009; Navin et al., 2011; Baslan et al., 2012; Ramskold et al., 2012; Picelli et al., 2013; Klein et al., 2015; Hashimshony et al., 2016; Baslan and Hicks, 2017; Zheng et al., 2017b) have been utilized in many cancer studies to achieve an understanding of tumors at the resolution of individual cells. In the present review, we focus mainly on recent cancer studies that have implemented single-cell RNA sequencing (scRNA-seq) and we summarize their relevant themes and results. Meanwhile, we discuss the therapeutic implications of these new knowledge and discoveries.

## INTRICATE TUMOR ECOSYSTEMS

**Figure 1:**
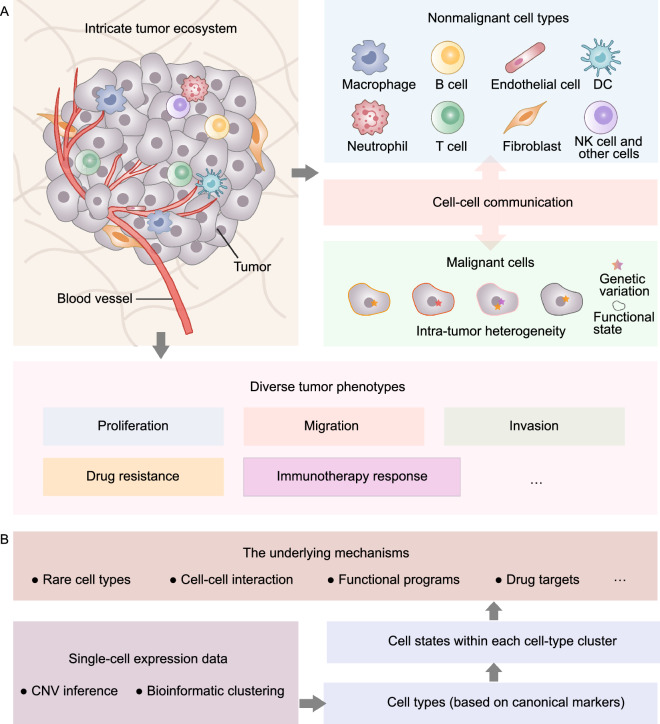
**Intricate tumor ecosystem and the workflow for scRNA-seq analysis of tumors.** (A) Tumors are complex ecosystems (left) composed of diverse malignant and nonmalignant cells (right). Nonmalignant cells include immune, endothelial, and stromal cells. Intra-tumor heterogeneity is an inherent feature of malignant cells mainly contributed by genetic heterogeneity and functional expression programs. These complicated cancer ecosystems underlie many critical facets of tumor biology. (B) Tumors are digested and sorted into single live cells and then profiled by scRNA-seq. Copy number variation (CNV) inference and canonical makers are used to annotate cell types. Cell states within each cell-type cluster are identified by bioinformatics clustering and functional programs. Advances in scRNA-seq have enabled a detailed dissection of tumor entities and enhanced our understanding of the underlying mechanisms at the resolution of individual cells

In a tumor, the diverse malignant cells, nonmalignant cells, and non-cellular factors interact synergistically to form a complicated “tumor ecosystem” that ultimately underlies many critical facets of tumor biology (Fig. 1A) (Suva and Tirosh, 2019). The tumor ecosystem mainly contains two parts: the tumor cells (i.e., malignant cells) and the tumor microenvironment (TME) (i.e., nonmalignant cells and non-cellular factors).

Regarding malignant cells, intra-tumor heterogeneity (ITH) is an inherent property influencing tumor growth, metastasis, recurrence, and response to therapies. Cell-to-cell expression ITH demonstrated by scRNA-seq can be interpreted at three levels (Tirosh and Suva, 2019): (1) genetic heterogeneity, in which subpopulations of tumor cells harbor subclonal mutations that are selected for during cancer evolution; (2) non-genetic heterogeneity, consisting of diverse epigenetic and differentiation programs; and (3) environmental and spatial factors related to the supply of nutrients, oxygen, and inter-cell interactions. Dissection of tumor architecture, especially on the expression level, can vastly enhance our knowledge of the molecular mechanisms related to the development and treatment resistance of cancer.

On the other hand, the TME plays a crucial role in shaping the tumor behavior. The TME comprises various immune cells (T cells, B cells, myeloid cells), stromal cells (fibroblasts, osteoblasts), other resident host cells (endothelial cells, adipocytes), and factors secreted by these cells, and non-cellular constituents of the extracellular matrix (ECM) (Maman and Witz, 2018). To describe the effect of the TME on tumor phenotype, models including the “seed-and-soil” hypothesis and the ecosystem networks are proposed (Amend et al., 2016; de Groot et al., 2017). These models highlight the effect of the TME components on regulating and remodeling tumor behavior. Multiple mechanisms are illustrated: (1) ligand–receptor crosstalk, Cells in the TME secrete multiple ligands that can be sensed by cancer cells or other cells, leading to the alteration of specific signaling pathways; (2) cell–cell interaction, such as the interaction between cytotoxic effector CD8^+^ T cells and tumor cells; and (3) cancer cells experiencing stress from the TME, such as hypoxia, starvation, and DNA damage (Maley et al., 2017). Furthermore, the TME contributes to the spatiotemporal inter- and intra-tumor heterogeneity. For example, there are three subclasses of tumor immune microenvironment that dictates tumor response to therapy: infiltrated–excluded, infiltrated–inflamed, and tertiary lymphoid structure (Binnewies et al., 2018). More importantly, different cell types and their diverse states in the TME can implement either anti-tumor or pro-tumor functions during cancer progression (Klein-Goldberg et al., 2014); thus, the entire tumor ecosystem should be considered when assessing the feasibility of a therapeutic approach.

Due to the complexity of the tumor ecosystem, analysis of a tumor at single-cell resolution, e.g., using scRNA-seq, is required. Benefiting from the high-throughput and unbiased gene expression analysis of scRNA-seq, not only cell types such as T cell, B cell, fibroblast, and tumor cell clusters, but also cell states within each cell-type (i.e., subclusters) can be identified in the tumor ecosystem (Fig. 1B) (Suva and Tirosh, 2019), by using the expression levels of canonical cell markers and/or genetic alterations such as large-scale copy number variations (Filbin et al., 2018). It is important to distinguish the cell states of the same cell type, because different gene expression of these states are related to cell cycle stage, different metabolic processes, and other dynamic programs. Moreover, rare or even new cell types with specific functional properties, such as stemness, inflammation, self-renewal, invasiveness, and drug resistance, can also be captured and characterized. Thus, precise identification of diverse cell types and states has both significant biological and clinical implications.

## CANCER CELL BIOLOGY ILLUMINATED BY scRNA-seq

In this section, we discuss research themes from recent scRNA-seq-based cancer studies that have linked heterogeneous expression programs of tumor subpopulations to clinical phenotypes.

### Tracing the cell type of tumor origin

Investigation of the initiation of tumor, e.g., identifying the types and states of cells from which malignancy originates, is an essential part of cancer research, because the cell type of tumor origin serves as a credible criterium for cancer classification. To identify the origin of cancer cells, prior knowledge of the cellular compositions and hierarchies of normal tissue is required (Vermeulen and Snippert, 2014). Single-cell RNA analysis enables deciphering of the constitution of normal tissues and subsequent lineage tracing. For example, in a recent scRNA-seq analysis, researchers found that tumor cells from distinct molecular subgroups of childhood cerebellar tumor mirror the transcriptional programs of different, temporally restricted cerebellar lineage cells (Vladoiu et al., 2019). They found that the Sonic Hedgehog medulloblastoma subgroup transcriptionally resembled the granule cell hierarchy, group 3 medulloblastoma mirrors Nestin (+) stem cells, group 4 medulloblastoma resembles unipolar brush cells, and PFA/PFB ependymoma and cerebellar pilocytic astrocytoma mirror prenatal gliogenic progenitor cells. Their results support the view that cerebellar tumors are a disorder from early brain development, revealing the origin of cerebellar cancer cells. In a similar study of renal cancer, investigators profiled 72,501 single-cell transcriptomes of human renal tumors and normal tissues from fetal, pediatric, and adult kidneys (Young et al., 2018). They found that cells from childhood Wilms tumors were matched with specific fetal cell types, and a canonical cancer transcriptome that matched a little-known subtype of proximal convoluted tubular cells in adult renal cell carcinoma was identified (Young et al., 2018). In another study, a human liver cell atlas was constructed (Aizarani et al., 2019), which has been used as a reference for the comparison of liver cancer cells. Through differential gene expression analysis, it was found that liver cancer cells lose the expression of cytochrome P450 genes, such as *CYP2E1* and *CYP2C8*, and the periportally zonated gene *CPS1*, and show increased expression of *AKR1B10*, which is related to hepatocellular carcinogenesis. More recently, Guo et al. characterized the prostate epithelial cell lineage hierarchy from mouse prostates using scRNA-seq (Guo et al., 2020). They identified a unique luminal cell type (termed type C luminal cell (Luminal-C)) marked by *Tacstd2*, *Ck4* and *Psca* expression. Functional experiments showed that Luminal-C cells which located at the distal prostate invagination tips had great capacity for organoid formation *in vitro* and prostate epithelial duct regeneration *in vivo*. They further identified the existence of Luminal-C cells in human prostate and suggested these cells can serve as the cellular origin of prostate cancer.

In summary, scRNA-seq is a powerful tool to delineate the cell types of tumor origin, and to identify their gene expression signatures and biomarkers.

### Developmental hierarchy and the cancer stem-cell model

Decades of studies provide evidence that tumor differentiation is controlled by a rare subset of cancer cells, termed “cancer stem cells” (CSCs) which possess many properties of stem cells (Dean et al., 2005; Clevers, 2011; Kreso and Dick, 2014). Similar to stem cells in normal tissues, CSCs are able to renew themselves and contribute to establish cellular hierarchies and heterogeneity of a tumor. Moreover, CSCs are thought to play important roles in therapy resistance and cancer relapse. Despite the cancer stem-cell model has been developed for decades (Hermann et al., 2007; Jan et al., 2012; Yamamoto et al., 2013; Lawson et al., 2015), the cellular states and putative markers of CSCs are far from clear in tumor samples.

scRNA-seq provides a unique avenue to uncover the cellular hierarchy of cancer cells and to identify CSCs. Using scRNA-seq, researchers found that primary glioblastoma cells display a spectrum of stemness and differentiation potential, revealing a putative CSC expression program (Patel et al., 2014). They also demonstrated that several transcription factors, such as POU3F2, NFIA, and NFIB, are important to regulate the stem-like phenotype. In other studies, researchers performed scRNA-seq on isocitrate dehydrogenase (IDH)-mutant oligodendroglioma (Tirosh et al., 2016b) and astrocytoma (Venteicher et al., 2017), revealing similar developmental hierarchies and lineages of glial differentiation of tumor cells. These two studies supported the cancer stem-cell model, in which the majority of cancer cells are well-differentiated and remain as oligodendrocyte-like or astrocyte-like cell lineages; however, a subset of undifferentiated cells possess properties of stem/progenitor cells. Of note, stem-like glioma cells with signatures of proliferation are enriched as the tumor grade increases, indicating that this minority group of cancer cells makes a large contribution to the growth and progression of IDH-mutant glioma. Furthermore, Filbin et al. demonstrated a similar cellular hierarchy and a subset of stem-like cells that resemble oligodendrocyte precursor cells (OPC-like) in primary H3K27M-glioma (Filbin et al., 2018), which constitute a higher fraction and exhibit greater tumor-propagating potential than their more differentiated counterparts. To investigate whether the cell state diversity within a tumor is determined by genetic factors, copy number variation (CNV) sub-clones inferred from scRNA-seq along with expression profiles were used to study the relationship between genetic subclones and intra-tumor cellular state diversity. For instance, in IDH1 or IDH2 mutant human oligodendrogliomas (Tirosh et al., 2016b), distinct CNV sub-clones displayed similar cellular hierarchies, suggesting that the cell states were primarily dictated by developmental programs. Consistent with this conclusion, an integrative and multi-omics study of glioblastoma (Neftel et al., 2019) revealed that the cellular hierarchy covered four cell states and these states were not strictly determined by CNV patterns. In glioblastoma, the CSC model was validated using functional experiments, which further explained why different cellular states remarkably converge to the same distribution of states in both patient-derived xenograft (PDX) models and patients (Neftel et al., 2019). In summary, scRNA-seq studies on gliomas support the cancer stem-cell model, implying that CSCs can act as potential targets for a better control of tumor differentiation and malignant progression.

In addition to gliomas, a subgroup of tumor cells displaying stem-like features are also found in other solid cancer types, such as prostate cancer (Horning et al., 2018), breast cancer (Savage et al., 2017), and hepatocellular carcinoma (Zheng et al., 2018). In prostate cancer, the advanced growth of the CSCs is related to attenuated androgen response and enhanced expression of cell cycle-related genes, which foster the plasticity of prostate cancer cells regarding their androgen dependence (Horning et al., 2018). In breast cancer, mesenchymal/stem-like tumor cells are present in EGFR inhibition responders (Savage et al., 2017). EGFR expression-high subpopulations show enhanced stem-like features, illustrating an EGFR-dependent hierarchy and enhancing the understanding of patient stratification for therapeutic intervention. As shown in a scRNA-seq analysis of hepatocellular carcinoma (Zheng et al., 2018), CSCs are phenotypically, functionally, and transcriptomically heterogeneous. Moreover, studies on the developmental hierarchies and the molecular signatures of stem cells of leukemia are instrumental for designing the molecularly targeted cancer therapies. In acute myeloid leukemia (AML), Velten et al. identified the gene expression programs of leukemic stem cells (LSCs) by combining scRNA-seq with lineage tracing using both nuclear and mitochondrial somatic variants (Velten et al., 2021). They highlighted that *FOS* and *CD96* may serve as important markers for LSCs. Meanwhile, Galen et al. provided an atlas of AML cell states and revealed the hierarchies related to disease progression (van Galen et al., 2019). The primitive AML cells were characterized by dysregulated transcriptional programs with co-expression of stemness and myeloid priming genes. Differentiated AML cells expressed diverse immunomodulatory genes and suppressed T cell activity *in vitro*. In chronic myeloid leukemia (CML), researchers identified distinct molecular signatures of CSCs and revealed their heterogeneity (Giustacchini et al., 2017). Notably, they uncovered a subgroup of CML stem cells with a distinct molecular signature that selectively persisted during prolonged TKI therapy. These therapy-resistant stem cells were characterized by marked quiescence-associated gene expression and showed dysregulation of specific genes and pathways (TGF-β, TNF-α, JAK-STAT, CTNNB1 and NFKB1A). These findings provide great insights into the cellular and molecular mechanisms of therapy resistance in CML.

Overall, scRNA-seq studies of tumors provide insight into the cellular states of CSCs and the molecular mechanisms of cellular hierarchy. However, the programs and biomarkers of CSCs should be further identified and verified by functional studies, which may contribute to the development of therapies targeting CSCs in tumor.

### Treatment resistance

A central problem in cancer therapy is that the majority of patients are unresponsive or develop acquired resistance. This issue can be experimentally interpreted using scRNA-seq to discover subsets of cancer cells with drug-susceptible or drug-resistant phenotypes, and to unravel their underlying programs. For example, a single-cell study in melanoma showed that two distinct transcriptional cell states, characterized by high levels of MITF expression and low MITF expression with elevated levels of AXL kinase, coexist in the same tumor (Tirosh et al., 2016a). The high-AXL program has been linked to intrinsic resistance to RAF and MEK inhibition, suggesting a subpopulation of high-AXL cells are involved in drug resistance and appear to increase upon treatment and recurrence. Rambow et al. reported that distinct drug-tolerant transcriptional states of single malignant cells were identified in the minimal residual disease of BRAF-mutant melanoma (Rambow et al., 2018). In this study, a neural crest stem cell transcriptional program, largely driven by the nuclear receptor RXRG, was found to mediate the development of resistance. In addition to resistance of targeted therapy, chemotherapy/radiotherapy resistance has also been studied by scRNA-seq. For instance, in nasopharyngeal carcinoma, a subset of malignant cells with an epithelial–immune dual feature was identified and found to be associated with poor prognosis (Jin et al., 2020). This clinical association is partly due to a higher expression level of genes (e.g., *STAT1*, *ISG15* and *IFIT1*) that are potentially related to DNA damage resistance and chemotherapy/radiotherapy resistance. Furthermore, similar studies regarding resistance to cancer immunotherapy were also performed. For example, Paulson et al. reported that two patients with metastatic Merkel cell carcinoma responded well at first, but finally developed late relapse, to the treatment with immune-checkpoint inhibitors and autologous Merkel cell polyomavirus-specific CD8^+^ T cells (Paulson et al., 2018). Using scRNA-seq, this study also identified dynamic transcriptional suppression of HLA-specific genes that can present the targeted viral epitope under intense CD8-mediated immunologic pressure, suggesting that the transcriptional suppression of Class I loci may underlie resistance to immunotherapies. Another study on melanoma tumors treated with immune checkpoint inhibitors (ICIs) provided a high-resolution landscape of the ICI-resistant cell state, which is associated with T cell exclusion and immune evasion (Jerby-Arnon et al., 2018). Recently, Miao et al. performed analysis on a model of squamous cell carcinoma treated with adoptive cytotoxic T cell transfer (ACT)-based immunotherapy, showing that TGF-β-responsive tumor-initiating stem cells are superior at resisting ACT (Miao et al., 2019). These cells selectively acquire CD80 (a surface ligand previously identified on immune cells) and CTLA4 to directly dampen cytotoxic T cell activity. In addition to the above-mentioned studies that demonstrated the mechanisms underlying adaptive and acquired resistance, other studies have identified subsets of circulating tumor cells (CTCs) with potential programs of drug resistance by comparing tumors prior to and following treatment (Patel et al., 2014; Miyamoto et al., 2015; Jordan et al., 2016).

### Metastasis

Cancer metastasis, the dissemination of tumor cells from the primary site and colonization in distant organs, causes the majority of cancer-related deaths. A series of scientific problems related to cancer metastasis, such as the variable dissemination abilities of tumor subpopulations and the transcriptional alterations involved in metastasis, as well as the different responses of the primary tumor and metastases to the same drug, can be explored using scRNA-seq. A recent study compared primary renal cell carcinoma and its lung metastases and found considerable variability in the activation of certain drug-target pathways (Kim et al., 2016). Just et al. compared primary ER^+^ breast cancer cells and their luminal UCD65 brain or luminobasal UCD46 liver metastases, and defined the unique molecular determinants of organ-specific metastasis (Just et al., 2018). Similarly, a study compared primary head and neck squamous cell carcinomas (HNSCCs) with their matched lymph node metastases, and showed that a partial epithelial-to-mesenchymal transition (p-EMT) program was spatially localized to the leading edge of each primary tumor, which was confirmed to be associated with metastases (Puram et al., 2017). These results suggested that the p-EMT signature may play a vital role in the initiation of metastatic dissemination but then disappear in the corresponding metastases. To understand the mechanism underlying metastasis, Chung et al. characterized the expression signatures and programs that promote metastatic progression in several rare subsets of primary triple-negative breast cancer using scRNA-seq, and identified strong EMT and stemness signatures, which may drive tumor progression and metastasis (Chung et al., 2017). This phenomenon was confirmed by another study, in which the potentially metastatic cells within migratory breast cancer cells exhibit overall signatures of EMT and stemness with variable expressions of marker genes (Pastushenko et al., 2018). Interestingly, a study on primary skin and mammary tumors identified different EMT stages in multiple tumor subpopulations, including intermediate hybrid states between completely epithelial and completely mesenchymal (Pastushenko et al., 2018; Chen et al., 2019). Functional experiments demonstrated that tumor cells with hybrid epithelial and mesenchymal phenotypes are more competent in reaching the circulation, colonizing the lungs, and forming metastatic sites. However, the molecular mechanisms underlying tumor metastatic dissemination and the detailed difference between primary tumors and metastases are still poorly understood, which requires further studies using scRNA-seq.

## TUMOR MICROENVIRONMENT PORTRAYED BY scRNA-seq: ONE CELL AT A TIME

It has been increasingly recognized that non-malignant cellular components of the TME, including diverse subsets of lymphocytes, myeloid cells, stromal cells, other resident host cells, and their secreted factors, have a significant impact on cancer progression and treatment outcomes (Chen and Mellman, 2017; Maman and Witz, 2018; Ren et al., 2018). Importantly, the TME can apply both pro- and anti-tumorigenic functions, which are contributed by various cell types and/or states as well as their dynamic changes. Therefore, it is important to characterize the various components of the TME and their dynamic changes, which may help to develop novel therapies. In this section, we review some recent progress in studying tumor-infiltrating lymphocytes, myeloid cells, and stromal cells using scRNA-seq.

### Tumor-infiltrating lymphocytes (TILs)

Within tumor-infiltrating immune cells, tumor-infiltrating lymphocytes (TILs), mainly comprised of T cells, B cells, and NK cells, can display both cytotoxic functions to directly eradicate malignant cells and pro-tumorigenic activities to facilitate the progression of cancer.

Among these TILs, tumor-infiltrating T cells are the major determinants of tumor behavior and the major responder to cancer immunotherapies, such as T cell immune-checkpoint blockade therapies (Chen and Mellman, 2017; Binnewies et al., 2018). Recently, the heterogeneous tumor-infiltrating T cells have been studied using scRNA-seq and their crucial roles have been understood from the following aspects.

First, multiple T cell subsets including rare/new cancer-associated subtypes have been systematically identified. Using scRNA-seq, the tumor-infiltrating T cell landscapes were characterized in head and neck cancer (Cillo et al., 2020), hepatocellular carcinoma (Zhang et al., 2019), rhabdoid tumors (Leruste et al., 2019), colorectal cancer (Zhang et al., 2018), breast cancer (Azizi et al., 2018; Savas et al., 2018), non-small-cell lung cancer (Guo et al., 2018), nasopharyngeal carcinoma (Jin et al., 2020), and melanoma (Tirosh et al., 2016a). For instance, Zheng et al. performed full-length scRNA-seq in 5,063 single T cells obtained from six hepatocellular carcinoma patients and identified 11 T cell subsets with different molecular and functional features (Zheng et al., 2017a). A similar study conducted by the same group depicted the landscape of infiltrating T cells in non-small cell lung cancer by analyzing 12,346 T cells from 14 patients, demonstrating 16 main T cell subsets including 7 CD8^+^ T cell subsets, 7 conventional CD4^+^ T cell subsets, and 2 regulatory T cell (Tregs) subsets (Guo et al., 2018). In a recent study depicting the tumor ecosystem of early-stage lung adenocarcinomas that present as subsolid nodules (SSNs), researchers compared the relative percentage of various T cell subsets in SSNs to those in normal lung tissue (nLung) and those in advanced-stage LUAD with metastasis (mLUAD) (Xing et al., 2021).

In addition to quantifying these well-known T cell subtypes, some rare functional clusters specific to the tumor context have been identified as well, because of the unprecedented resolution offered by scRNA-seq. For example, a CD8^+^ tissue-resident memory T cell subset, distinct from conventional T effector memory cells, was found in breast cancer (Savas et al., 2018). In detail, high expression levels of immune checkpoints (such as TIM3, PD1, CTLA4, TIGIT, and LAG3) and effector proteins (including granzyme B and perforin) were observed in these cells. Moreover, the identified gene signature of this T cell subset is significantly associated with improved survival in triple-negative breast cancer, providing improved prognostic information. In another example, a newly defined T cell subset in colorectal cancer, a group of CXCL13^+^BHLHE40^+^ TH1-like cells, was found to be enriched in microsatellite-unstable tumors (Zhang et al., 2018). These cells express a high level of IGFLR1, which shows a co-stimulatory function to enhance the production of IFN-γ, and also exhibit high clonal expansion and proliferation. This finding may explain why patients with microsatellite-instable colorectal tumors respond well to immune checkpoint blockade, and may offer a new therapeutic target in immune therapy.

Secondly, the functional state transition and lineage connection of distinct tumor-infiltrating T cell subtypes have been deciphered: T cells can enter into different states with distinct functional behaviors. For instance, effector T cells can suppress tumor progression by lysing cancer cells, whereas dysfunctional or exhausted effector T cells with elevated expression of inhibitory receptors (e.g., PD-1, TIM-3, CTLA-4, LAG3, and TIGIT) impair antitumor effector functions (Zhang and Zhang, 2019). Using scRNA-seq, dysfunctional states of tumor-infiltrating T cells and their expression signatures and regulators have been characterized in several studies (Singer et al., 2016; Tirosh et al., 2016a; Zheng et al., 2017a; Guo et al., 2018; Zhang et al., 2018, 2019). Moreover, the dynamic relationships among different T cell states were also unraveled. For example, a model of continuous T cell activation was identified in breast cancer (Azizi et al., 2018), in addition to a model of dysfunction with an underlying gene module uncoupled from T cell activation (Singer et al., 2016). Furthermore, scRNA-seq combined with single-cell TCR analysis has given further insight into the developmental connection among T cell states. Using both techniques, Zhang et al. demonstrated that the CD4^+^GZMK^+^ memory T cells have a lineage connection with CXCL13^+^ TH1-like cells in colorectal cancer, suggesting these two subsets have developmental relationship (Zhang et al., 2018). Similarly, exhausted T cells have developmental connections with GZMK^+^ T cells, a subtype related to effector T cells, in both liver and colorectal cancers (Zheng et al., 2017a; Zhang et al., 2018, 2019). Two “pre-exhausted” T cell subsets including CD8^+^GZMK^+^ and CD8^+^ZNF683^+^ T cells have been identified in non-small-cell lung cancer (Guo et al., 2018). In accordance with the continuous transition model of exhaustion, a recent study in human melanoma identified a gradient of T cell dysfunction, demonstrating a separation between bystander cytotoxic T cells and a transitional-to-dysfunctional lineage (Li et al., 2019). This study also showed that, surprisingly, the exhausted T cells are highly proliferative and clonal and their related dysfunctional signature was shown to be linked to the tumor reactivity of the T cell pool. These results facilitate the understanding of the regulatory mechanisms underlying T cell state transition and the identification of new immunotherapy targets.

Thirdly, the features of treatment-response expression of T cells and their changes have been depicted, which provide new insights into the T cell response to treatment. For example, by analyzing CD8^+^ T cell pools prior to and following combined Tim-3^+^ and PD-1-blockade treatment, an induced shift from naive-like to memory precursor-like and effector-like PD-1-CD8^+^ T cell subsets was identified (Kurtulus et al., 2019). Moreover, the transcriptional regulator Tcf7/Tcf1 was found to be necessary for maintaining memory-precursor T cells and the efficacy of immunotherapies. Another study analyzed T cells from patients with basal or squamous cell carcinoma prior to and following anti-PD-1 therapy and showed that the response to therapy depends on the newly recruited T cells rather than the reinvigoration of pre-existing T cells (Yost et al., 2019). Recently, Bi et al. characterized the single-cell transcriptomes of cancer and immune cells from advanced renal cell carcinoma patients before or after immune checkpoint blockade treatment (Bi et al., 2021). They found that CD8^+^ T cell phenotypes were reprogrammed to express higher levels of co-inhibitory receptors (PDCD1, TIGIT, and HAVCR2) and effector molecules (GZMB, PRF1, and IFNG) in responders.

In addition to T cells, among the TILs, tumor-infiltrating B cells and NK cells are also active regulators of tumor progression and treatment response. Increasing evidence has shown that tumor-infiltrating NK cells can function in both anti-tumor cytotoxic and ineffective states (Sceneay et al., 2012; Brodbeck et al., 2014), and some tumor-infiltrating B cell populations exhibit pro-malignancy functions (Affara et al., 2014; Pylayeva-Gupta et al., 2016). Recently, the diverse subtypes and gene signatures of tumor-infiltrating B cells and NK cells have been characterized in several cancer types using scRNA-seq (Tirosh et al., 2016a; Chung et al., 2017; Lavin et al., 2017; Zhang et al., 2019; Cillo et al., 2020). For instance, in head and neck squamous cell carcinoma (HNSCC), 11 B cell subsets were identified, and they presented distinct distributions between HPV− and HPV+ HNSCCs (Cillo et al., 2020): HPV+ HNSCCs contain a greater number of B cells which are more in non-germinal center states than HPV− HNSCC. Moreover, two other scRNA-seq studies highlighted the important role of tumor-infiltrating B cells in mediating tumor response to therapies. In detail, Hollern et al. created novel mammary tumor models and revealed that T cell activation mediated by B cells and antibodies generation are essential in immunotherapy response (Hollern et al., 2019). Another study identified an emerging ICOSL^+^ B cell subset after neoadjuvant conventional chemotherapy (Lu et al., 2020), and showed that the opposite functions of B cells during chemotherapy were shaped by complement signals and determined by CD55 expression, providing important implications for future anti-tumor therapies.

### Tumor-infiltrating myeloid cells (TIMs)

In addition to TILs, tumor-infiltrating myeloid cells (TIMs) also play an important role in shaping tumor behavior. The diverse TIMs, mainly tumor‐associated macrophages (TAMs), dendritic cells (DCs), and neutrophils, possess different and dynamic anti- or pro-tumorigenic functions (Binnewies et al., 2018; Maman and Witz, 2018), directly interacting with tumor cells and can modify the activities of other cells (Salmon et al., 2016). Recently, extensive heterogeneity among TIMs has been revealed by delineating the landscape of the major cell types and states of TIMs in head and neck squamous cell carcinoma (Cillo et al., 2020), hepatocellular carcinoma (Zhang et al., 2019), melanoma (Li et al., 2019), rhabdoid tumors (Leruste et al., 2019), non-small cell lung cancer (Song et al., 2019), and breast cancer (Azizi et al., 2018) using scRNA-seq. For example, in human melanoma, multiple subsets of TIMs containing macrophages, monocytes, DCs, plasmacytoid DCs, and a small population of osteoclast-like cells were identified (Li et al., 2019). Similarly, in breast cancer, subsets of TIMs are also complicated and were delineated in four branches in the cell activation diffusion map (Azizi et al., 2018): one branch almost totally covers TAMs from three subsets; two branches capture a gradual trace from blood monocytes to intra-tumoral monocytes; and one branch distinguish a plasmacytoid DC subset from other monocyte subsets. Different from the above cancers, the composition of TIMs in rhabdoid tumors (genomically simple pediatric cancer) is simple, which was identified as DCs, monocytes, and TAMs (Leruste et al., 2019).

Among TIMs, TAMs show great functional and molecular plasticity, so that TAMs are a hot research topic in cancer research. It has been known from conventional studies that distinct cellular states of TAM have positive or negative roles in cancer. For example, M1-macrophages are considered to possess anti-tumor functions by phagocytosing and producing Th1‐promoting cytokines, such as IL‐12, IL‐23, and TNF‐α, while M2‐macrophages are thought to have pro-tumor functions by regulating angiogenesis, suppressing immune surveillance, promoting inflammation, and stimulating cancer cell motility by secreting growth factors and chemokines (IL‐10, TGF‐β, PD‐L1, and ARG‐1) (Engblom et al., 2016). Recently, more extensive heterogeneity among TAMs was revealed using scRNA-seq. For instance, TAM subsets with distinct molecular and functional features were comprehensively identified in rhabdoid tumors (Leruste et al., 2019); there are a subset of macrophages expressing *APOE, FOLR2, RNASE1*, and the genes defining macrophage activation signature, a subset of macrophages expressing *MMP9*, cathepsin K, and proteases relating to tissue remodeling, and a subset of macrophages expressing *HMOX*, an enzyme with anti-inflammatory properties. Of note, the genes related to angiogenesis, immunosuppressive functions, and M2-like signature are significantly enriched in the APOE^+^ macrophages. Furthermore, scRNA-seq identifies a controversial issue whether TAMs conform to the polarization model in different cancers. In lung cancer, macrophages show rheostatic phenotypes and become M2-polarized in tumors, suggesting their detrimental activities in the TME (Lambrechts et al., 2018). However, TAMs in breast cancer (Azizi et al., 2018), liver cancer (Zhang et al., 2019), and gliomas (Muller et al., 2017) frequently co-express M1 and M2 gene signatures in individual cells, arguing a more complicated pattern than the classical M1/M2 model. These studies suggested that further analysis of the differential trajectories and functional identification of TAMs should be performed. Similar to macrophages, DCs have also been demonstrated to exhibit adverse functions in the TME (Song et al., 2019; Zhang et al., 2019; Cillo et al., 2020). For instance, a subset of tumor-infiltrating LAMP3^+^ DCs was identified in liver cancer and was shown to express various immune-relevant ligands with the potential ability to regulate multiple subtypes of lymphocytes (Zhang et al., 2019). Different from the dual character of macrophages and DCs, neutrophils have been shown that certain types always exert pro-tumor functions (Maman and Witz, 2018). For instance, using scRNA-seq, neutrophils were found to improve cell cycle progression of circulating tumor cells, which increased the probability of cancer metastasis (Szczerba et al., 2019).

### Stromal cells

As a vital part of the TME, non-parenchymal cells of the stroma in the tumor niche, mainly fibroblasts and osteoblasts, exert notable effects on many hallmarks of cancer. Tumor associated stromal cells regulate the behaviors of tumor cells in several ways, such as remodeling and degrading the extracellular matrix (ECM) by secreting collagen and proteolytic enzymes; promoting EMT in tumor cells; recruiting or regulating immune cell infiltration; and releasing cytokines, chemokines, and growth factors (Orimo and Weinberg, 2006; Maman and Witz, 2018; Valkenburg et al., 2018). Although the active role of stromal cells within tumors is already appreciated, the detailed repertoire in different cancer types remained elusive until recently. For example, a systematic census of stromal cells in lung cancer was provided using scRNA-seq (Lambrechts et al., 2018), in which a total of 53 stromal cell subtypes including novel subpopulations were characterized. Similarly, a cellular taxonomy of bone marrow stroma in homeostasis and leukemia was characterized using scRNA-seq (Baryawno et al., 2019), in which 17 stromal subsets with distinct hematopoietic regulatory genes covering new mesenchymal, pericyte, fibroblast, and endothelial subsets were identified. This study also showed that impaired mesenchymal osteogenic differentiation and reduced regulatory molecules are necessary for normal hematopoiesis to occur in leukemia. These findings deepen our understanding of stromal cells perturbed by malignancy.

The dominant constituents of the tumor stroma cells are cancer-associated fibroblasts (CAFs), which always harbor pro-malignancy properties and are often referred to as myofibroblasts due to their altered shape and expression of ACTA2 and prolyl endopeptidase FAP (Micallef et al., 2012). The CAFs can proliferate, migrate, and secrete ECM factors and abnormal patterns of collagen (Kalluri, 2016). Recently, the heterogeneity of CAFs in different cancer types and their contributions to diverse cancer cell phenotypes have been revealed by several scRNA-seq studies (Li et al., 2017; Puram et al., 2017; Bartoschek et al., 2018; Lambrechts et al., 2018; Ligorio et al., 2019; Dominguez et al., 2020). Accompanying the single-cell landscape of CAFs, novel CAF subtypes with special molecular and functional properties have been identified. For example, Dominguez et al. uncovered a subset of CAFs that are programmed by TGF-β and express LRRC15 (Dominguez et al., 2020), which were confirmed to surround tumor islets in patients with pancreatic cancer and correlate with poor response to anti-PD-L1 therapy. Moreover, cross-species single-cell analysis of pancreatic ductal adenocarcinoma identified a new population of antigen-presenting CAFs expressing MHC class II and CD74 but not classic co-stimulatory molecules (Elyada et al., 2019). In an animal model system, antigen-presenting CAFs were shown to have the capability of presenting antigens to CD4^+^ T cells and modulating the immune response. These new subsets of CAFs offer non-immune targets for combinatorial therapy in the clinical setting. However, the heterogeneity of other stromal cells and their functional features still remain unclear and deserve further exploration at the single-cell level.

## CONCLUDING REMARKS

Cancer is a highly heterogeneous and dynamic disease. Bulk expression profiling of clinical specimens has facilitated our understanding of tumor biology and clinical stratification. However, tumors are increasingly viewed as intricate ecosystems, in which heterogeneous tumor cells interact with diverse immune, endothelial, and stromal cells. The precise characterization of diverse cell types and intra-tumor heterogeneity has been masked by bulk analyses. The rapid development in scRNA-seq technology has laid the foundation for deciphering tumors at single-cell resolution. Here, we review recent studies using scRNA-seq to address the scientific questions in cancer biology. These studies have revolutionized our understanding of cancer biology by uncovering the cellular landscape of tumors, identifying rare subtypes and their underlying cellular programs, and characterizing the interaction between tumor cells and their microenvironment.

**Figure 2:**
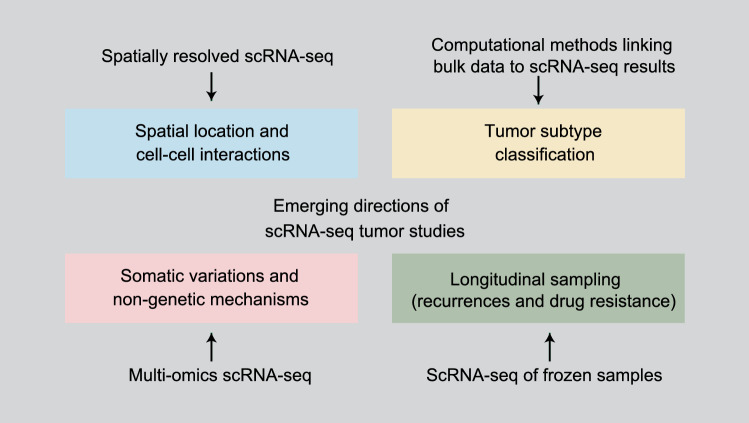
Emerging directions of cancer studies and development of scRNA-seq technologies

Although great progress has been made in this field, more attention should be paid to the emerging challenges and future directions (Fig. 2). First, the molecular capture efficiency of scRNA-seq technology and bioinformatics approaches should be improved. Secondly, multi-omics technologies require further development to combine mRNA expression analysis with other molecular features such as protein expression levels and DNA methylation. Thirdly, spatially resolved single-cell technology will be a powerful tool to predict cell–cell interactions and provide spatial heterogeneity information. The studies discussed in this review are the inception of single-cell tumor profiling. In the future, further analyses will shed light on cancer biology and lay the foundation for novel therapeutic management.

## ABBREVIATIONS

AML, acute myeloid leukemia; CML, chronic myeloid leukemia; CNV, copy number variation; CSCs, cancer stem cells; ECM, extracellular matrix; ITH, intra-tumor heterogeneity; LSCs, leukemic stem cells; NGS, next-generation sequencing; PDX, patient-derived xenograft; scRNA-seq, single-cell RNA sequencing; TME, tumor microenvironment
